# Retinoic Acid Drives Aryl Hydrocarbon Receptor Expression and Is Instrumental to Dioxin-Induced Toxicity during Palate Development

**DOI:** 10.1289/ehp.1003075

**Published:** 2011-08-01

**Authors:** Hugues Jacobs, Christine Dennefeld, Betty Féret, Matti Viluksela, Helen Håkansson, Manuel Mark, Norbert B. Ghyselinck

**Affiliations:** 1 Institut de Génétique et de Biologie Moléculaire et Cellulaire (IGBMC), Centre National de la Recherche Scientifique (CNRS UMR7104), Institut National de la Santé et de la Recherche Médicale (INSERM) U964, Université de Strasbourg, Illkirch, France; 2 Department of Environmental Health, National Institute for Health and Welfare, Kuopio, Finland; 3 Institute of Environmental Medicine, Karolinska Institutet, Stockholm, Sweden; 4 Hôpital Universitaire de Strasbourg, Illkirch, France

**Keywords:** AHR, cleft, mesenchyme, mouse, nasal epithelium, RAR, retinaldehyde dehydrogenase (RALDH), teratogenesis, 2,3,7,8-tetrachlorodibenzo-*p*-dioxin (TCDD)

## Abstract

Background: Palate development depends on complex events and is very sensitive to disruption. Accordingly, clefts are the most common congenital malformations worldwide, and a connection is proposed with fetal exposure to toxic factors or environmental contaminants, such as dioxins. There is increasing evidence that dioxin interferes with all-*trans*-retinoic acid (atRA), a hormone-like signal derived from vitamin A, which plays an essential role during embryonic development. Although similarities have been described between dioxin-induced toxicity and the outcome of altered atRA signaling during palate development, their relationship needs to be clarified.

Objectives: We used a genetic approach to understand the interaction between atRA and dioxin and to identify the cell type targeted by dioxin toxicity during secondary palate formation in mice.

Methods: We analyzed the phenotype of mouse embryos harboring an atRA-sensitive reporter transgene or bearing null mutations for atRA-synthesizing enzymes (RALDH) or atRA receptors (RAR) and maternally exposed to 2,3,7,8-tetrachlorodibenzo-*p*-dioxin (TCDD) at gestation day 10.5.

Results: We found that an intact atRA signal was required to enable TCDD to induce cleft palate. This mandatory atRA signal was generated through the activity of RALDH3 in the nasal epithelium and was transduced by RARγ (RARG) in the nasal mesenchyme, where it notably controlled aryl hydrocarbon receptor (*Ahr*) transcript levels. TCDD also did not alter the developmental pattern of atRA signaling during palate formation.

Conclusions: TCDD-induced alteration of secondary palate development in the mouse appears to depend on atRA signaling, which controls AHR expression. This mechanism is likely conserved throughout vertebrate evolution and may therefore be relevant in humans.

Embryonic development of the mammalian face relies on a sequence of complex and interdependent molecular, cellular, and tissue interactions. Disruption of these developmental processes often results in orofacial defects, which are the most common of all congenital disorders in humans. The second most frequent nonsyndromic orofacial defect is isolated cleft palate, which has a frequency of approximately 1:2,500 in European newborns (Cobourne 2004). Its effect on speech, hearing, appearance, and cognition leads to long-lasting adverse outcomes impairing social integration (Vallino et al. 2008). Isolated cleft palate is currently considered to have a multifactorial etiology in which a deleterious genetic background is combined with environmental factors. Gene linkage and association studies indicate that the pathogenesis of nonsyndromic isolated cleft palate relies on a combination of multiple mutations in different genes (Lidral et al. 2008), and epidemiological studies have revealed a correlation between increased risk for oral cleft and exposure to dioxin-like chemicals during pregnancy (Leite et al. 2002; Murray 2002). In laboratory animals, notably in mice, exposure to 2,3,7,8-tetrachlorodibenzo-*p*-dioxin (TCDD) during organogenesis causes cleft palate (Courtney and Moore 1971; Couture et al. 1990). Because developing palatal shelves of human, rat, and mouse respond similarly to TCDD exposure in organ cultures (Abbott et al. 1999; Couture et al. 1990), the mouse is an ideal *in vivo* model for studying teratogenic effects of dioxins.

At the molecular level, TCDD alters gene expression by activating the aryl hydrocarbon receptor (AHR). Unliganded AHR resides in the cytoplasm in an inactive complex with heat-shock molecules. Binding of TCDD to AHR causes the dissociation of the receptor from the complex and its translocation into the nucleus, where it dimerizes with the AHR nuclear translocator (ARNT). The AHR/ARNT heterodimer functions as a transcriptional activator by binding to specific DNA sequences called dioxin response elements (DREs) located in the regulatory regions of AHR-responsive genes (Beischlag et al. 2008; Pohjanvirta et al. 2011). Mice with a homozygous ablation of the *Ahr* gene suffer from various age-related pathologies; this suggests that AHR exerts important physiological functions (Fernandez-Salguero et al. 1995). Thus, understanding the molecular mechanisms through which TCDD exposure results in a cleft palate may provide clues not only to the mechanisms of TCDD teratogenicity but also to the nature of homeostatic AHR functions.

There is increasing evidence that environmental pollutants such as dioxin-like compounds interfere with all-*trans*-retinoic acid (atRA) signaling (Novák et al. 2008). atRA is a pleiotropic, paracrine or autocrine signaling molecule produced from vitamin A through oxidative reactions carried out by the cytosolic retinaldehyde dehydrogenases RALDH1, RALDH2, and RALDH3 (Duester 2008). atRA acts as a hormone by binding to and activating α, β, and γ isotypes of atRA receptors (RARs; RARA, RARB, RARG, respectively), which belong to the nuclear hormone receptor superfamily and function as ligand-dependent transcription factors interacting with regulatory regions located in target genes. In a large variety of tissues, RARs act by forming heterodimers with one of the rexinoid receptors, retinoid X receptor (RXR; α, β, or γ isotypes), which bind 9-*cis* but not atRA (Mark et al. 2006). Similarities between dioxin toxicity and atRA deficiency or excess have often been pointed out (Nilsson and Håkansson 2002; Novák et al. 2008). Accordingly, atRA excess induces a cleft palate (Abbott et al. 1989), as does TCDD exposure (Courtney and Moore 1971; Couture et al. 1990). In many instances, the effects of TCDD on atRA-controlled processes *in vitro* appear to be mediated by AHR either interfering positively or negatively with atRA signaling in certain cell types or changing activity of the enzymes responsible for transformation of retinoids (Novák et al. 2008). However, further investigation is needed to confirm that the mechanisms shown to operate *in vitro* are indeed mediating TCDD-induced defects *in vivo*.

In this study, we used mouse embryos harboring null mutations in the genes coding for RALDH3, RARA, or RARG to unravel the possible interaction between atRA and TCDD during palate development and to reassess the etiology of TCDD-induced cleft palate. We demonstrate that TCDD does not alter the pattern of atRA signaling in the embryonic face. However, we show that an atRA signal generated through the activity of RALDH3 in the nasal epithelium and transduced by RARG in the nasal mesenchyme is mandatory to enable TCDD to induce cleft palate when administered at gestation day (GD) 10.5, notably through controlling the levels of *Ahr* expression. In addition, our results suggest that TCDD acts not directly on the developing palatal shelves, but on the mesenchyme adjacent to the nasal epithelium.

## Materials and Methods

*Animal use.* Mice were housed in an animal facility licensed by the French Ministry of Agriculture (agreement B67-218-5). Animal experiments were supervised by one of the authors who is qualified for experimenting with mice, in compliance with the European legislation on care and use of laboratory animals (agreement 67-205). The mice were treated humanely and with regard for alleviation of suffering.

*Mice genotyping and treatments.* The transgenic line *Tg(RARE-Hspa1b/lacZ)12Jrt* and the lines carrying the *Ahr^tm1Bra^*-, *Rara^tm3.1Ipc^*-, *Rarg^tm1Ipc^*-, and *Aldh1a3^tm1.1Pcn^*-null alleles were genotyped as previously described (Chapellier et al. 2002; Dupé et al. 2003; Lohnes et al. 1993; Rossant et al. 1991; Schmidt et al. 1996). Noon of the day a vaginal plug was observed was considered GD0.5. At GD10.5, pregnant mice were given a single dose of 100 mg/kg atRA (Biomol International, Plymouth Meeting, PA, USA) or 30 μg/kg TCDD (Wellington Laboratories, Guelph, ON, Canada) dissolved in sunflower oil (Sigma, Lyon, France) by oral gavage. The number of fetuses and litters analyzed and an overview of cleft palate occurrence as a function of treatments and genotypes are presented in Supplemental Material, Table 1 (http://dx.doi.org/10.1289/ehp.1003075).

*Phenotype analysis.* We stained skeletons with Alcian blue and Alizarin red as previously described (Lufkin et al. 1992). For detection of β-galactosidase activity, we performed 5-bromo-4-chloro-3-indolyl-beta-d-galacto-pyranoside (XGal)-based staining (Rossant et al. 1991) and embryos were postfixed in Bouin’s fluid, embedded in paraffin, serially sectioned, and then counterstained with eosin. Whole-mount *in situ* RNA hybridization was performed as previously described (Wendling et al. 2001). *In situ* hybridization and immunohistochemistry on cryosections were also performed as previously described (Vernet et al. 2006), using embryos that were fixed for 1 hr in 4% (wt/vol) phosphate-buffered paraformaldehyde at 4°C.

*RNA analysis.* We prepared transverse slices of the nasopalatal region from GD11.5 embryos (*n* ≥ 3 for each condition) from which the eyes and the maxillary component of first branchial arches were removed. Wild-type (WT) or RAR-deficient (*Rara*^–/–^/*Rarb*^–/–^/*Rarg*^–/–^) mouse embryonic fibroblasts (MEFs) were maintained as previously described (Epping et al. 2007). At subconfluency, we added cycloheximide (10^–6^ M) 1 hr before atRA (10^–6^ M) and harvested cells 6 hr after atRA. We extracted total RNA using Trizol reagent (Invitrogen, Life Technologies, Villebon-sur-Yvette, France), converted it to cDNA, and analyzed it by real-time polymerase chain reaction performed in a Realplex Mastercycler (Eppendorf, Le Pecq, France). We normalized the transcript levels relative to that of *Rplp0* (ribosomal protein, large, P0) transcript (MGI:1927636), whose expression is not altered in mutant mice or in atRA- or TCDD-treated fetuses. We analyzed each sample in triplicate and assessed results using Student’s *t*-test.

## Results

**Figure 1:**
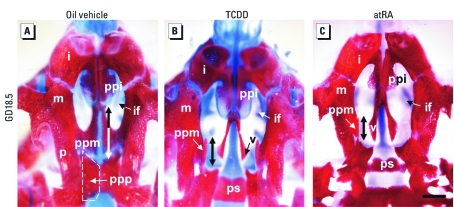
TCDD and atRA similarly impair palate development: ventral view of palatal regions of GD18.5 WT fetuses treated at GD10.5 with oil vehicle (*A*), TCDD (*B*), and atRA (*C*). Bone was stained with Alizarin red and cartilage with Alcian blue. Both TCDD and atRA induced cleft palates through which one can see the presphenoid and vomer bones. The arrows indicate the length of the palatal processes of maxillary bones. Abbreviations: i, incisive bone; if, incisive foramen; m, maxillary bone; p, palatine bone; ppi, palatal process of incisive bone; ppm, palatal process of maxillary bone; ppp, palatal process of palatine bone; ps, presphenoid bone; v, vomer bone. Bar = 1 mm.

*TCDD administration and excess atRA at GD10.5 induce identical cleft palates.* To compare the morphological outcomes of TCDD and atRA treatments on palatal development, we analyzed skeletons of 34 GD18.5 fetuses. An oral dose of TCDD (30 μg/kg) to pregnant WT mice at GD10.5 always (*n* = 27 fetuses) inhibited the development of the palatal processes of the maxillary bones, which were hypoplastic, as well as those of the palatine bones, which were agenic (Figure 1B). In contrast, other parts of these bones (e.g., alveolar, orbital, and zygomatic processes) were normal [see Supplemental Material, Figure 1 (http://dx.doi.org/10.1289/ehp.1003075)]. Treatment of pregnant WT mice with atRA (100 mg/kg) at GD10.5 also systematically induced a cleft palate (*n* = 7 fetuses), which was indistinguishable from its TCDD-induced counterpart (Figure 1C; see also Supplemental Material, Figure 1) and was not accompanied by other craniofacial defects. Therefore, both TCDD exposure and atRA excess at GD10.5 induce a cleft palate through inhibition of palatal shelf development. This finding raised the possibility either that atRA activates AHR or that TCDD mimics the effects of atRA excess through activating this pathway.

**Figure 2:**
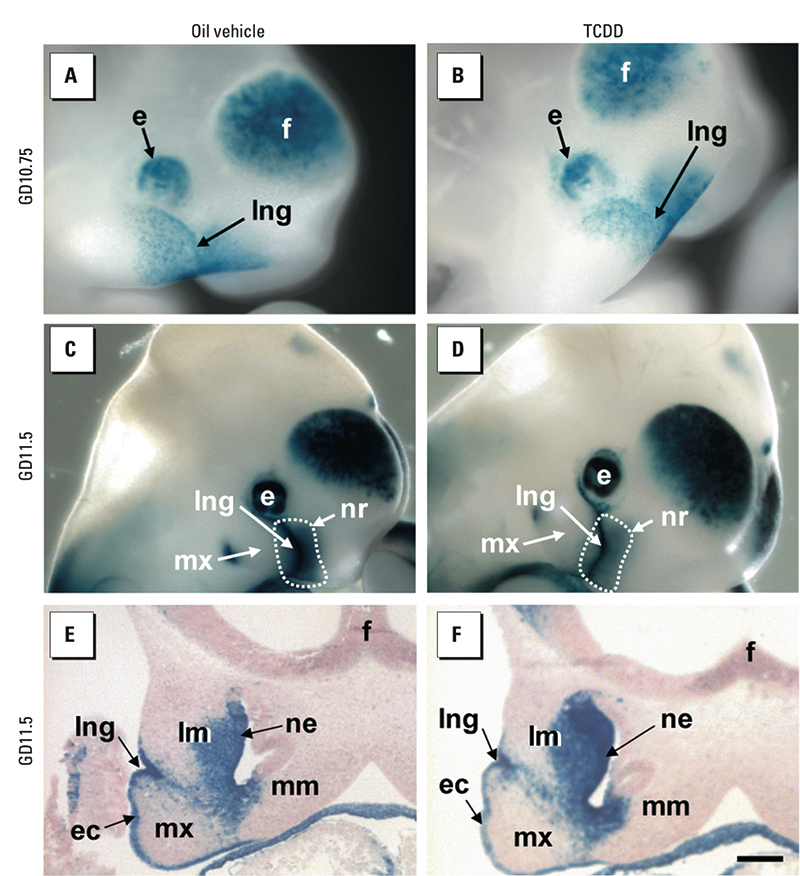
TCDD does not modify the pattern of endogenous atRA signaling. *Tg(RARE-Hspa1b/lacZ)12Jrt* transgenic embryos were treated at GD10.5 with vehicle (*A*,*C*,*E*) or TCDD (*B*,*D*,*F*), and the outcome on atRA signaling was analyzed after 6 hr (*A*, *B*) and 24 hr (*C*–*F*). *A*–*D* are external views, and *E* and *F* are frontal histological sections of embryos after XGal staining (blue) to reveal *Tg(RARE-Hspa1b/lacZ)12Jrt* activity, which indicates atRA-responsive cells. Vehicle- and TCDD-treated fetuses show identical patterns of atRA activity, indicating that the dynamics and extent of atRA signaling remain unaltered upon TCDD treatment. Abbreviations: e, eye; ec, ectoderm; f, forebrain; lm, lateral mesenchyme of the frontonasal region; lng, lacrimonasal groove; mm, medial mesenchyme of the frontonasal region; mx, maxillary prominence of first branchial arch; ne, nasal epithelium; nr, nasal region. Bar in *F* = 250 μm for *A* and *B*, 360 μm for *C* and *D,* 200 μm for *E* and *F*.

**Figure 3:**
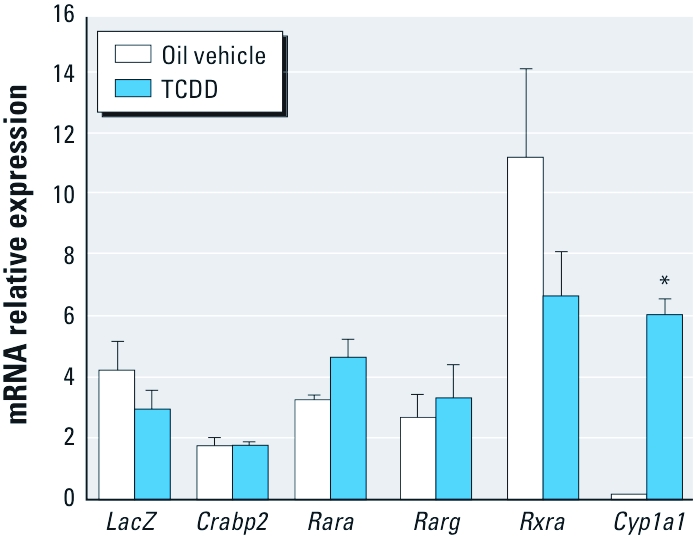
TCDD does not affect atRA-dependent gene expression, shown by relative mRNA levels for atRA target genes (*LacZ*, *Crabp2*, *Rara*, *Rarg*, and *Rxra*) and a TCDD-target gene (*Cyp1a1*) in GD11.5 nasopalatal regions of *Tg(RARE-Hspa1b*/*lacZ)12Jrt* embryos treated with vehicle or TCDD. Data are mean + SD of triplicates from three nasopalatal regions in each experimental condition. *p < 0.05.

*Excess atRA does not produce cleft palate through activating AHR, and reciprocally, TCDD does not modify the developmental pattern of atRA signaling in the face.* To test for these hypotheses, we first intercrossed *Ahr*^+/–^ mice and then fed the pregnant females atRA at GD10.5 and examined their progeny at GD18.5. We observed a cleft palate in all *Ahr*^–/–^ fetuses (*n* = 5), thus ruling out the possibility that atRA was activating AHR to induce a cleft palate [see Supplemental Material, Figure 2 (http://dx.doi.org/10.1289/ehp.1003075)]. Then, to test whether TCDD activated atRA signaling, we analyzed its effects on embryos harboring the *Tg(RARE-Hspa1b/lacZ)12Jrt* transgene (Rossant et al. 1991), which is used to monitor variations in atRA signaling (Duester 2008). We analyzed its activity at GD10.75, GD11.5, and GD12.5 (*n* = 5 embryos at each developmental age) and found that control and TCDD-treated fetuses displayed identical patterns of XGal staining in the frontonasal region (Figure 2), indicating that the developmental pattern of atRA signaling remained unaltered upon TCDD treatment. To extend these observations, we quantified the mRNA levels of *lacZ* and endogenous atRA-target genes, including *Crabp2* (cellular retinoic acid binding protein), *Rara*, and *Rarg* (Balmer and Blomhoff 2002). In agreement with the lack of effect of TCDD on transgene activity, we did not detect a significant difference for any of these four genes in the nasopalatal region of TCDD-treated (*n* = 3) or control (*n* = 3) embryos. In addition, we verified that *Rxra* mRNA level was not altered, ruling out the possibility that TCDD acted through modulating expression of the RAR partner (Figure 3). Importantly, we confirmed the efficiency of TCDD treatment by the induction of *Cyp1a1* (cytochrome P450 1A1), a well-established target gene of TCDD-activated AHR (Abbott et al. 1999). Together, these results demonstrate that the ability of TCDD to induce a cleft palate cannot be accounted for by increased or ectopically activated atRA signaling.

**Figure 4:**
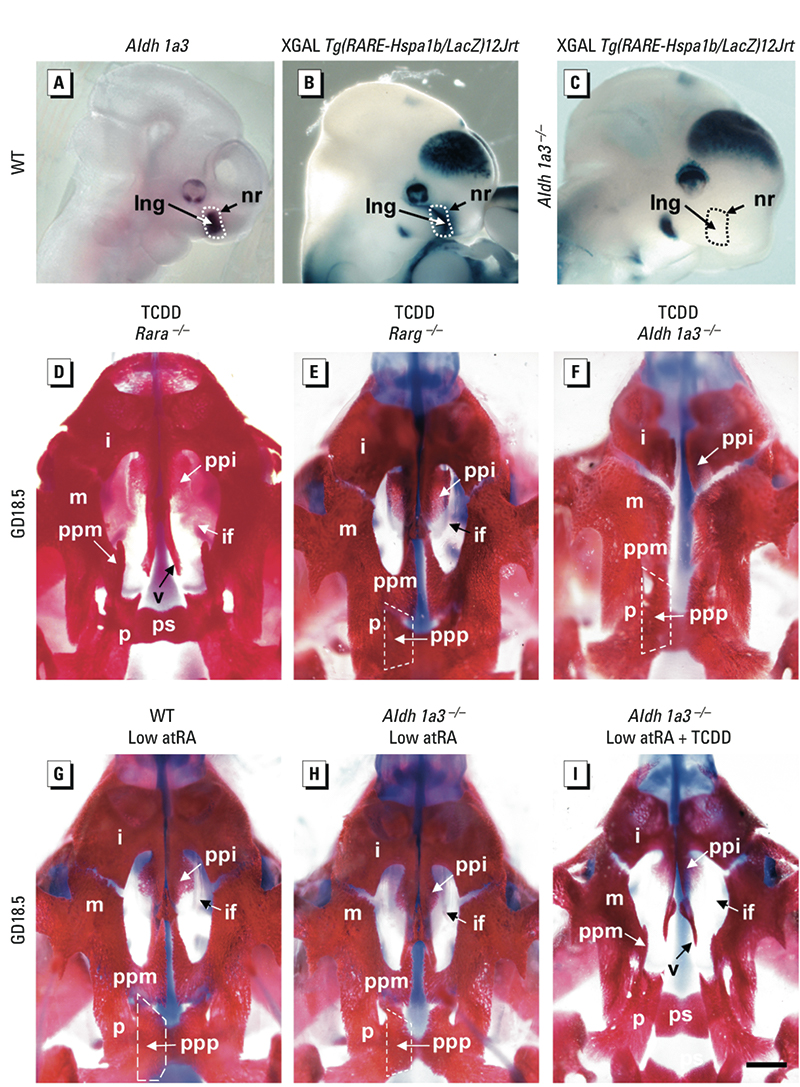
TCDD is unable to induce cleft palate when atRA signaling is impaired. (*A*) Whole-mount *in situ* hybridization indicates restriction of *Aldh1a3* expression to the nasal epithelium region. (*B* and *C*) XGal staining (blue) reveals *Tg(RARE-Hspa1b/lacZ)12Jrt* transgene activity in WT and *Aldh1a3*^–/–^ embryos, illustrating the requirement of only RALDH3 for atRA synthesis in the nasal region. (*D*–*I*) Ventral view of palatal regions (Alizarin red/Alcian blue staining) of GD18.5 fetuses treated at GD10.5 with TCDD. *Rara*^–/–^ mutants (*D*) displayed cleft palate, whereas *Rarg*^–/–^ (*E*) and *Aldh1a3*^–/–^ (*F*) mutants were resistant to this malformation. Administration of low doses of atRA (2 mg/kg every 12 hr from GD8.5 to GD12.5) failed to induce cleft palate in WT (*G*) and *Aldh1a3*^–/–^ mutant (*H*) embryos but was sufficient to restore TCDD toxicity for palate development in *Aldh1a3*^–/–^ mutants (*I*). Abbreviations: i, incisive bone; if, incisive foramen; lng, lacrimonasal groove; m, maxillary bone; nr, nasal region; p, palatine bone; ppi, palatal process of incisive bone; ppm, palatal process of maxillary bone; ppp, palatal process of palatine bone; ps, presphenoid bone; v, vomer bone. Bar in *I *= 300 μm for *A*–*C* and 1 mm for *D*–*I*.

*TCDD is unable to induce a cleft palate when atRA signaling is impaired.* The above results did not exclude the possibility that endogenous, atRA-dependent events are required to allow TCDD-induced inhibition of palatal shelf development. Between GD10.5 and GD12.5, we detected atRA signals in the frontonasal region but not in the palate per se (Figure 4B). During this period of development, RALDH3 is the sole RA-synthesizing enzyme expressed in the nasopalatal region as assessed by *in situ* hybridization (Figure 4A) and by the complete disappearance of *Tg(RARE-Hspa1b/lacZ)12Jrt* activity in *Aldh1a3*^–/–^ fetuses (Figure 4C). RARA and RARG are the sole nuclear receptors driving atRA activity during craniofacial development (Lohnes et al. 1994). To test the possibility that TCDD relies on endogenous atRA to induce cleft palate, we impaired atRA signaling *in vivo* through ablation of genes coding for atRA receptors or atRA-synthesizing enzymes. Thus, mutant mice carrying *Rara*-, *Rarg*-, or *Aldh1a3*-null alleles were mated, and we dosed pregnant females at GD10.5 with TCDD or vehicle [see Supplemental Material, “Material and Methods” (http://dx.doi.org/10.1289/ehp.1003075)]. We analyzed skulls from GD18.5 null mutants (i.e., *Rara*^–/–^, *Rarg*^–/–^, and *Aldh1a3*^–/–^) and control WT littermates (*n* ≥ 3 for each genotype and treatment). All *Rara*^–/–^ mutants exposed to TCDD consistently displayed a cleft palate (Figure 4D). In contrast, the palates of TCDD-treated *Rarg*^–/–^ and *Aldh1a3*^–/–^ mutants were invariably closed (Figure 4E,F). Analysis of dissected bones indicated that the palatal processes of maxillary and palatine bones developed normally in *Aldh1a3*^–/–^ mutants (see Supplemental Material, Figure 1). Most important, the ability of TCDD to induce cleft palate was restored in *Aldh1a3*^–/–^ embryos rescued for atRA signaling through the repeated administration of a low dose of atRA (2 mg/kg), which was not otherwise teratogenic for palate development (Figure 4G–I). This demonstrates that atRA is the missing signal impairing the action of TCDD in the *Aldh1a3*-null genetic background. Thus, our results show that inhibition of palatal shelf development by TCDD actually requires atRA-dependent signal(s) generated through the activity of RALDH3 and mediated by RARG.

**Figure 5:**
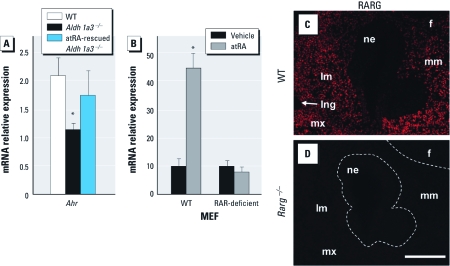
AHR expression depends upon RALDH3 in the frontonasal region. (*A* and *B*) Relative *Ahr* mRNA levels in GD11.5 nasopalatal regions of WT, *Aldh1a3*^–/–^ and atRA-rescued (2 mg/kg) *Aldh1a3*^–/–^ embryos (*A*) and in WT or RAR-deficient MEFs treated with vehicle or atRA (*B*). Data are the mean + SD of triplicates from at least three samples (nasopalatal regions or cell cultures) in each experimental condition. (*C* and *D*) Immunohistochemistry showing RARG protein localization in the mesenchyme surrounding the nasal epithelium in WT embryo (*C*). An *Rarg*^–/–^ embryo was used as a negative control for immunostaining (*D*). Abbreviations: f, forebrain; lm, lateral mesenchyme of the frontonasal region; lng, lacrimonasal groove; mm, medial mesenchyme of the frontonasal region; mx, maxillary prominence of first branchial arch; ne, nasal epithelium. Bar = 200 μm. *p < 0.05 in ligand-treated versus vehicle-treated samples.

*AHR expression depends on RALDH3 in the frontonasal region.* The most straightforward explanation for the above-mentioned results would be that expression of AHR relies upon atRA synthesized by RALDH3 within or close to the developing palate. To test for this possibility, we compared expression of *Ahr* in the nasopalatal regions isolated from GD11.5 WT (*n* = 3) and *Aldh1a3*^–/–^ (*n* = 3) embryos. We found a significantly decreased steady-state level of *Ahr* mRNA in mutants lacking RALDH3. Importantly, *Ahr* mRNA was restored to normal levels in *Aldh1a3*^–/–^ embryos (*n* = 3) rescued for atRA signaling through the repeated administration of low, nonteratogenic doses of atRA (Figure 5A). To further assess the effect of atRA on *Ahr* expression, we examined the level of *Ahr* mRNA in WT MEFs cultured with cycloheximide, an inhibitor of protein translation, and found a 5-fold increase upon atRA stimulation (Figure 5B). This indicated that atRA-activated RAR controlled AHR expression without the need for intermediate protein synthesis, suggesting a direct effect of RAR on *Ahr* gene. Accordingly, atRA did not increase *Ahr* mRNA levels in RAR-deficient MEFs (Figure 5B). Together, these results support the idea that *Ahr* expression requires atRA generated by RALDH3 and can explain why palate formation was unaffected by TCDD treatment in *Aldh1a3*^–/–^ mutants.

Together, our findings that *a*) RARG, but not RARA, is mandatory for TCDD to induce cleft palate and *b*) RAR directly controls *Ahr* gene expression imply that the AHR-expressing cells at the origin of the malformation are necessarily distributed within the domain where RARG is operational. Using immunohistochemistry, we found that RARG was expressed in the mesenchyme adjacent to nasal epithelium (Figure 5C,D) and overlapped with an active atRA signaling only in the mesenchyme distributed lateral to the nasal epithelium (compare Figures 2E and 5C). Thus, TCDD likely acts through activating AHR present in the mesenchyme localized lateral to the nasal epithelium.

## Discussion

Several experiments underline the impact of TCDD on retinoid homeostasis, but whether it yields states of functional vitamin A deficiency or excess remains a matter for further clarification. Overall, findings on TCDD-induced growth retardation, abnormal immune function, and developmental defects reminiscent of vitamin A–deficient states suggest that dioxin-like compounds reduce atRA signaling (Murphy et al. 2007). Several mechanisms supporting this scenario have been proposed. First, TCDD exposure mobilizes retinyl esters from liver stores and may thus rapidly exhaust them (Nilsson and Håkansson 2002). Second, TCDD may impair RAR functioning as it inhibits both atRA binding and induction of target genes in cultured cells (Lorick et al. 1998; Weston et al. 1995). Third, TCDD may enhance atRA catabolism through inducing enzymes such as CYP1A1 and CYP2S1 that can transform atRA into less active metabolites (Lampen et al. 2000; Smith et al. 2003).

An opposite set of data suggests that dioxin-like compounds may enhance atRA signaling, particularly with regard to bone and developmental defects (Nilsson and Hakansson 2002). Different scenarios have been proposed to explain the enhancement of atRA signaling by TCDD. First, TCDD may increase atRA synthesis (Schmidt et al. 2003) because DRE have been identified in *Aldh1a2* gene coding for RALDH2, the most potent atRA synthesizing enzyme (Wang et al. 2001) and because AHR-induced CYP1A1 can participate in atRA synthesis (Chen et al. 2000; Tomita et al. 1996). Second, *in vitro* experiments indicate that TCDD induces expression of *Rarg* and *Rrxa* (Murphy et al. 2004) and that activated AHR is able to divert RARΑ from its co-repressor, thereby allowing the receptor to become transcriptionally active in the absence of atRA (Widerak et al. 2006).

Regarding development of the secondary palate, the present study demonstrates that TCDD and excess atRA share similar teratogenic properties when administered at GD10.5 as their effects are morphologically indistinguishable from one another, as previously reported (Abbott et al. 1989; Moore et al. 1973). Furthermore, a combination of low doses of each compound synergistically induces this defect (Birnbaum et al. 1989). Thus, an interaction exists between dioxin-induced and atRA-dependent events involved in this developmental defect. The possibility that a decrease in atRA signaling could account for TCDD-induced effects appears unlikely because the palatal malformations generated upon functional vitamin A deficiency or *Rar* gene ablations *a*) affect essentially the development of the primary palate and *b*) are never isolated, but instead occur as part of a holoprosencephaly syndrome (Lohnes et al. 1994). These features are clearly distinct from those of dioxin-induced cleft palates. We therefore explored the possibility that TCDD exposure during gestation may enhance atRA signaling through increasing either atRA levels or RAR activity. Our experiments using mice harboring an atRA-sensitive reporter transgene, as well as the quantitative analysis of atRA-target gene expression in the palatal region, revealed that TCDD does not modify the pattern of atRA signaling in the facial region during palate development.

In contrast, because *Aldh1a3*^–/–^ and *Rarg*^–/–^ mutants are resistant to TCDD-induced clefts, our study provides evidence that a functional, intact atRA signal originating from RALDH3, which is the sole atRA-synthesizing enzyme expressed in the palatal region and mediated by RARG in the facial mesenchyme, is required for TCDD to exert teratogenic effects on palate development. Accordingly, we provide evidence that the atRA signaling pathway is instrumental in AHR signaling in the palatal region, notably through controlling *Ahr* transcript levels [see Supplemental Material, Figure 3 (http://dx.doi.org/10.1289/ehp.1003075)]. Even though no atRA-response element is characterized in this gene, we show a direct involvement of atRA-activated RAR in this process. Interestingly, a role of atRA and RAR in the control of AHR expression has been previously demonstrated in medaka fish (Hayashida et al. 2004). Because RARs exert their functions heterodimerized with RXRA during mouse development (Mark et al. 2006), it is likely that RARG acts with RXRA to control AHR expression. This hypothesis, however, cannot be confirmed because *Rxra*^–/–^ mutants die before the completion of palate closure (reviewed in Mark et al. 2006). Nonetheless, our finding that *Rxra* expression was unchanged after TCDD exposure and the fact that RXRA is not activated by atRA (Duester 2008) together support the view that RXRA plays no major role aside from its partnership with RARG. Importantly, the fact that *Ahr*^–/–^ mice developed cleft palates upon atRA excess allows us to rule out the possibility that atRA binds to and activates AHR, as opposed to other retinoids (Gambone et al. 2002; Soprano and Soprano 2003; Soprano et al. 2001). We also excluded the possibility that TCDD acts through binding to, and activating, RARG because ablation of *Aldh1a3* was sufficient to prevent TCDD-induced cleft palate.

AHR expression has been detected in the developing mouse at several sites and distinct time points (Abbott et al. 1995). In addition, the temporal and spatial context of AHR activation after TCDD exposure *in vivo* has been determined in a transgenic mouse model (Willey et al. 1998). Together, these studies, which focused mainly on elevation and fusion of palatal shelves, have highlighted roles of AHR in mesenchyme and epithelium at GD14.5 but did not identify the cell type(s) that were targeted by TCDD at GD10.5. Interestingly, our study indicates that AHR is required at GD10.5 in the mesenchyme lateral to the nasal epithelium. Therefore, at the beginning of palatogenesis, TCDD does not act directly on nascent, first-arch–derived palatal shelves, but rather on the frontonasal mesenchyme distributed lateral to the nasal epithelium.

## Conclusions

Our results indicate that atRA-activated RARG controls the expression of AHR at GD10.5 in the developing palate, which in turn appears necessary for TCDD to induce cleft palate. TCDD, however, does not alter the pattern of atRA-signaling in the developing face. These findings provide evidence about the molecular mechanism through which TCDD exposure at GD10.5 can result in a cleft palate and thereby clarify a possible mechanism of action for TCDD. The etiology of cleft palate induced by TCDD at later developmental stages (e.g., GD12.5) may stem from another mechanism (Abbott and Birnbaum 1990; Birnbaum et al. 1989). Nonetheless, because both AHR and RAR are universal signaling systems conserved across vertebrate species, including humans (Campo-Paysaa et al. 2008; Hahn 2002), it is possible that the mechanistic findings in the present study are of general relevance. Accordingly, *Ahr* expression is controlled by atRA-activated RAR both in mouse (present study) and in medaka fish (Hayashida et al. 2004). In this context, intact atRA signaling may be mandatory to enable the AHR message not only in the developing palate at GD10.5 but also in other organ systems.

## Supplemental Material

(1.1 MB) PDF
