# Supplementary material for: Retinoic Acid Drives Aryl Hydrocarbon Receptor Expression and Is Instrumental to Dioxin-Induced Toxicity during Palate Development
Source: Environ Health Perspect. 2011 Aug 1;119(11):1590–5. doi: 10.1289/ehp.1003075 (PMC3226489; doi:10.1289/ehp.1003075)
Supplement: (1.1 MB) PDF [file ehp.1003075.s001.pdf]

Supplemental Material

Retinoic Acid Drives Aryl Hydrocarbon Receptor Expression and is Instrumental to Dioxin-Induced Toxicity during Palate Development

Hugues Jacobs, Christine Dennefeld, Betty Féret, Matti Viluksela, Helen Hakansson, Manuel Mark and Norbert B. Ghyselinck

Supplemental Material, Material and Methods .... page 2

Supplemental Material, Table 1 .... Page 5

Supplemental Material, Figure 1 .... page 6

Supplemental Material, Figure 2 .... page 7

Supplemental Material, Figure 3 .... page 8

Supplemental Material, References .... page 9

Supplemental Material, Material and Methods.

Mice, treatments and genotyping. All mice used in the present study were backcrossed with C57BL/6 mice to reach a genetic background estimated to 99.9% C57BL/6. Heterozygote mice (i.e., *Ahr*$^{+/-}$, *Rara*$^{+/-}$, *Rarg*$^{+/-}$ or *Aldh1a3*$^{+/-}$ mice) were inter-crossed to generate mutant (i.e., *Ahr*$^{-/-}$, *Rara*$^{-/-}$, *Rarg*$^{-/-}$ or *Aldh1a3*$^{-/-}$) and wild-type (WT) control (i.e., *Ahr*$^{+/+}$, *Rara*$^{+/+}$, *Rarg*$^{+/+}$ or *Aldh1a3*$^{+/+}$) progenies. Mice bearing the *Tg(RARE-Hspa1b/lacZ)12Jrt* transgene were crossed with C57BL/6 mice. On the one hand, atRA (Biomol International, 99.85% purity) was first dissolved in ethanol (2% of final volume), and then diluted with sunflower oil (Sigma) to the final concentration of 0.5mg/ml for treating with 2mg/kg, or 10mg/kg for treating with 100mg/kg. On the other hand, TCDD solution (HPLC grade, Wellington Laboratories, purity > 99% dissolved in nonane) was evaporated in a fume hood overnight, and subsequently dissolved in sunflower oil (Sigma) to 3µg/ml final concentration (stock solution). The time-point for treatment was selected to completely cover the development of palatal shelves, which starts as outgrowth of the maxillary palatal process on E11. TCDD has been shown to distribute fast to the fetus after oral maternal dosing in mice and rats (Abbot et al. 1996; Hurst et al. 1998; Hurst et al. 2000). TCDD is detected in the embryo as soon as 30 min after maternal dose and the peak embryonic tissue concentrations are reached within 8–24 h (depending on the time points selected for the study), after which the tissue levels are fairly constant for several days. Similarly, atRA has been shown to reach mouse embryo rapidly (within 2 hours) and efficiently (Creech Kraft et al., 1989). Rescue of atRA deficiency in *Aldh1a3*$^{-/-}$ embryo was achieved by maternal gavage with atRA at 2mg/kg, administered every 12 hr from E8.5 to E12.5 (Dupé et al. 2003). Cleft palate induction by atRA was achieved by a single maternal gavage with atRA at 100mg/kg, administered at E10.5. Cleft palate induction by TCDD was achieved by a single maternal gavage with TCDD at 30µg/kg, administered at E10.5. Importantly, administration of the sunflower oil vehicle to pregnant

females never yielded a cleft palate, while responsiveness of the *Tg(RARE-Hspa1b/lacZ)12Jrt* transgenic mice to TCDD was confirmed by the observation of a cleft palate in all E18.5 exposed fetuses. Embryos and fetuses were collected by caesarean section, and the yolk sacs were taken for DNA extraction. We performed genotyping of *Rara*, *Aldh1a3* and *Rarg* alleles by PCR as described (Chapellier et al. 2002, Dupé et al. 2003; Lohnes et al. 1993). In the case of *Ahr*, we used primers 5'-TCTTGGGCTCGATCTTGTGTC-3' and 5'-TTGACTTAATTC CTTCAGCGG-3' to amplify a 500bp-long fragment from the WT allele, whereas we used primers 5'-TTGGGTGGAGAGGCTATTCG-3' and 5'-AGGTGAGATGACAGGAGATC-3' to amplify a 150bp-long fragment from the null allele. In the case of *lacZ*, we used primers 5'-CGCCGACGGCACGCTGATTG-3' and 5'-GTTTCAATATTGGCTTCATC-3' to amplify a 300bp-long fragment from the transgene.

Immunohistochemistry. We performed detection of RARG on cryosections from embryos that were fixed for 1 hour in 4% (w/v) phosphate-buffered paraformaldehyde at 4°C, embedded in Tissue-Tek® O.C.T. compound and frozen on dry ice (Vernet et al 2006). After rinsing three times for 5 min at room temperature in phosphate-buffered saline containing 0.1% Tween 20 (v/v), we incubated the sections in a humidified chamber for 16 h at 4°C with the affinity-purified anti-RARG RPγ(mF) antibody (Rochette–Egly et al. 1991) diluted at 1/500. Detection of the bound primary antibodies was achieved for 45 min at room temperature, using a Cy3-conjugated goat anti-rabbit IgG (Biomol Immuno Research Laboratories, Exeter, UK) diluted at 1/1000. To assess antibody specificity, we used sections from *Rarg*$^{-/-}$ mutants as negative controls.

Gene expression analysis. The samples for mRNA levels analyses were immediately frozen in liquid nitrogen, and stored at –80°C until use. Total RNA were extracted using Trizol reagent (Invitrogen). Aliquots of 1µg total RNA were converted to cDNA using a QuantiTect Reverse Transcription Kit and according to manufacturer's instructions (Qiagen). Quantitative

analysis of cDNA was then carried out by real-time PCR performed in a Realplex Mastercycler (Eppendorf) using QuantiTect SYBR Green PCR Kits, and according to manufacturer's instructions (Qiagen). Conditions were 50 cycles with denaturation for 15 sec at 95°C, annealing for 15 sec at 60°C, and elongation for 20 sec at 72°C. The transcript levels were normalized relative to that of *Rplp0* transcript (MGI:1927636), which is responsive neither to atRA (Roy et al. 1995; Taneja et al., 1997; Rochette-Egly et al. 2000; Goelden et al, 2005; Matt et al. 2005) nor to TCDD (Hanlon et al. 2005). The primers were 5'-ACCAGAACTGTGAGGGTTGG-3' and 5'-CTCCCATCGTATAGGGAGCA-3' for *Ahr*, 5'-CACTGGAGCTAGGAGGCAAG-3' and 5'-GTACAGCACTGGCCTTGGTT-3' for *Aldh1a3*, 5'-ACCCTGAAGTGCTCGACATC-3' and 5'-AGGAAGGCCTTGACCTTTTC-3' for *Rplp0*, 5'-ACCCTGAAGTGCTCGACATC-3' and 5'-AGGAAGGCCTTGACCTTTTC-3' for *Crabp2*, 5'-CAGCCTTCCCAAATGGTTTA-3' and 5'-GCCTGGGCTACACAAGAC TC-3' for *Cyp1a1*, 5'-ACTATCCCGACCGCCTTACT-3' and 5'-TAGCGGCTGATGTTGA ACTG-3' for *lacZ*, 5'-TCTCCCTGGACATTGACCTC-3' and 5'-TCATTGTGTCTTGCT CAGGC-3' for *Rara*, 5'-GATCTGGTTACCCAGGAGCA-3' and 5'-GGCTTATAGACCC GAGGAGG-3' for *Rarg*. 5'-AACTCTCCAACGGGTCGAGGC-3' and 5'-GCTGGGGGCT ACCAGTCCCG-3' for *Rxra*.

Cell culture. WT mouse embryonic fibroblasts (MEF), as well as MEF bearing null mutations for all three RAR genes (*Rara*$^{-/-}$/*Rarb*$^{-/-}$/*Rarg*$^{-/-}$), were kindly provided by Dr Hinrich Gronemeyer (IGBMC). Cells were maintained at 37°C and 5% CO2 in DMEM containing 4.5g/l glucose and antibiotics, and supplemented with 10% charcoal-treated fetal calf serum. At 70-80% confluency we added cycloheximide (10^{-6}M) in the medium one hour prior to adding atRA (10^{-6}M) for 6 hours. At this point, cells were scraped, centrifuged, frozen dry in liquid nitrogen and stored at –80°C until use.

Supplemental Material, Table 1.

Genotype of pregnant mother	Genotype of father	Treatment	Number of litters analysed	Total of embryos or fetuses	Number of embryos with the indicated genotype, and percentage of them displaying cleft palate	
					+/+	–/–
WT	WT	atRA (100mg/kg) at E10.5	1	7	7 (100%)	–
Ahr$^{+/–}$	*Ahr*$^{+/–}$	atRA (100mg/kg) at E10.5	2	16	5 (100%)	5 (100%)
WT	WT	TCDD (30µg/kg) at E10.5	6	27	26 (96%)	–
Tg(RARE-Hspa1b/lacZ)12Jrt	WT	TCDD (30µg/kg) at E10.5	1	8	4 (100%)	–
Rara$^{+/–}$	*Rara*$^{+/–}$	TCDD (30µg/kg) at E10.5	2	12	3 (100%)	3 (100%)
Rarg$^{+/–}$	*Rarg*$^{+/–}$	TCDD (30µg/kg) at E10.5	3	14	4 (100%)	3 (0%)
Aldh1a3$^{+/–}$	*Aldh1a3*$^{+/–}$	TCDD (30µg/kg) at E10.5	3	14	3 (100%)	4 (0%)
Aldh1a3$^{+/–}$	*Aldh1a3*$^{+/–}$	atRA (2mg/kg twice a day from E8.5 to E12.5)	3	24	3 (0%)	5 (0%)
Aldh1a3$^{+/–}$	*Aldh1a3*$^{+/–}$	atRA (2mg/kg twice a day from E8.5 to E12.5) + TCDD (30µg/kg) at E10.5	2	22	4 (100%)	3 (100%)

All mice used in the present study were estimated with a genetic background being 99.9% C57BL/6. WT, wild-type (C57BL/6).

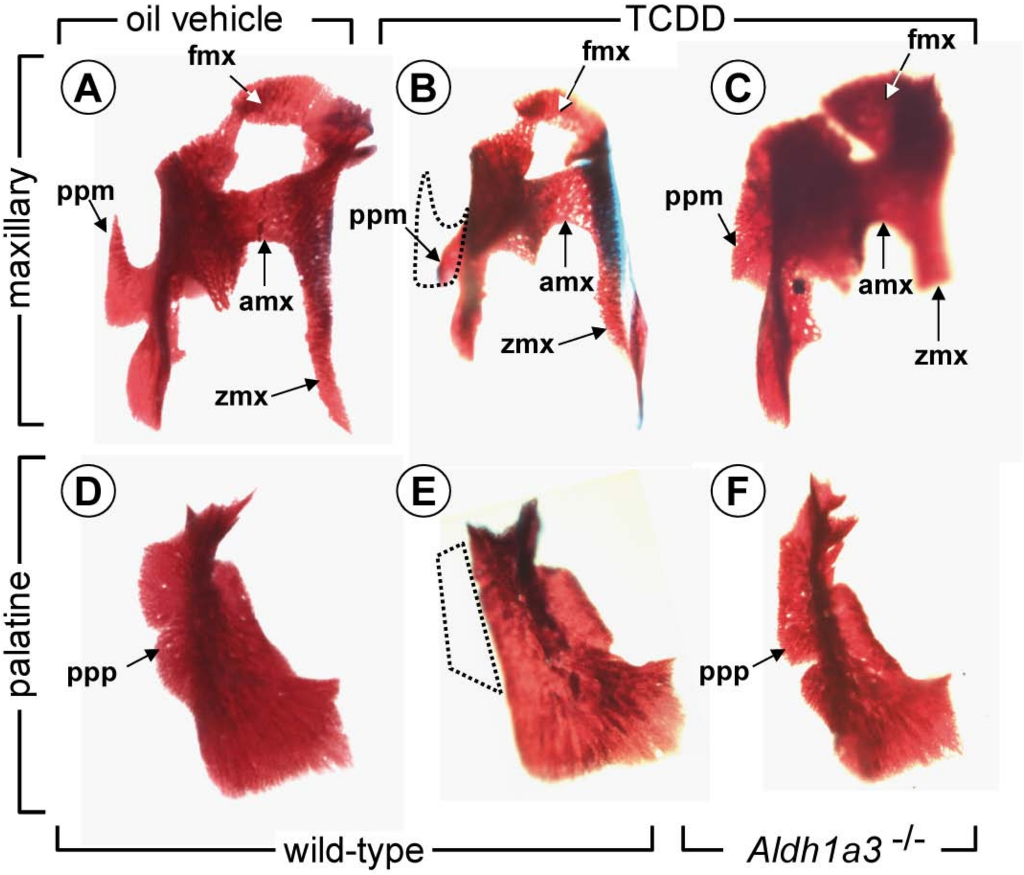

Supplemental Material, Figure 1. TCDD alters the shape of maxillary and palatine bones. Maxillary (A-C), and palatine (D-F) bones dissected from 18.5 WT (A, B, D, E) and *Aldh1a3*$^{-/-}$ mutant (C, F) fetuses treated at E10.5 with vehicle (A,D) or TCDD (B, C, E, F). Bone is stained by alizarin red. (B) TCDD induces the hypoplasia of the palatal process of maxillary bone in WT fetuses. (C) In contrast, the same treatment failed to induce hypoplasia of the palatal process of maxillary bone in *Aldh1a3*$^{-/-}$ mutants. (E, F) TCDD-induced agenesis of the palatal process of the palatine bone in WT fetuses (E) is never observed in *Aldh1a3*$^{-/-}$ mutant mice (F). The black dotted lines (in B, E) encompass missing structures. Bones were photographed at the same magnification. amx: alveolar process of maxillary bone; fmx: frontal process of maxillary bone; ppm: palatal process of maxillary bone; ppp: palatal process of palatine bone; zmx: zygomatic process of maxillary bone.

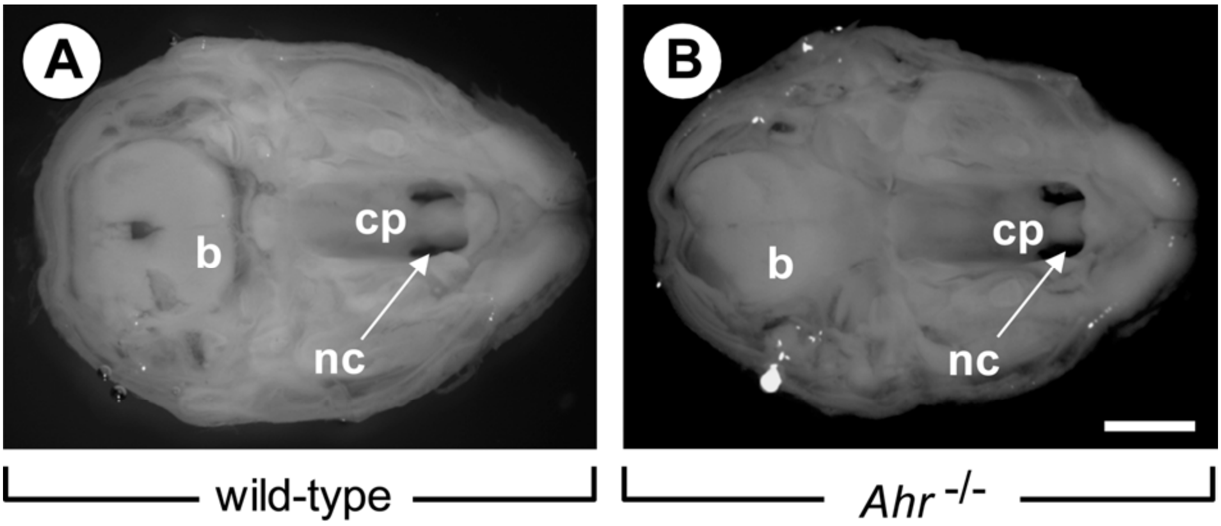

Supplemental Material, Figure 2. *Ahr*$^{-/-}$ mutants are sensitive to atRA-induced cleft palate. (A) Ventral view of heads from E18.5 *Ahr*$^{-/-}$ mutant (left) and WT littermate (right) treated with atRA at E10.5. Lower jaw and tongue were removed. Mutant and WT fetuses both display a cleft palate, indicating that atRA was not activating AHR to induce a cleft palate. b: brainstem; cp: cleft palate; nc: nasal cavity. Scale bar: 2mm.

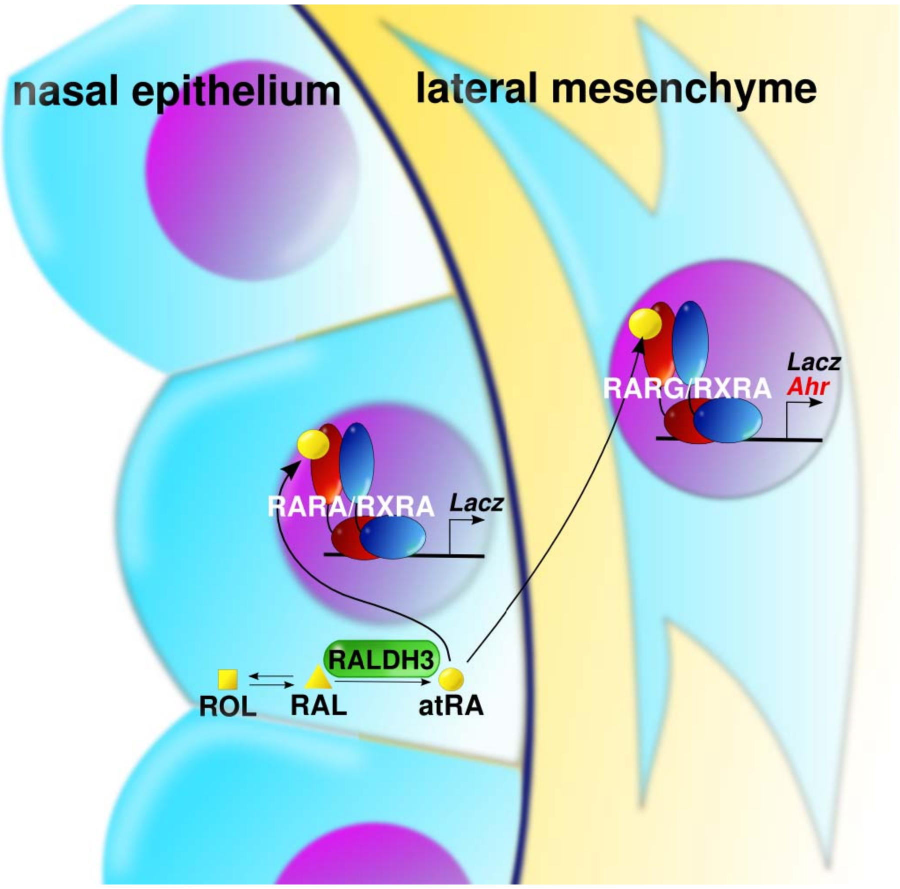

Supplemental Material, Figure 3. Model schematizing the action of atRA in the control of *Ahr* expression in the naso-palatal region during embryogenesis. The cells in which an active atRA signal operates were identified through the use of atRA-responsive *LacZ* reporter transgene (blue cells), while the cells in which RALDH3 and RARG are expressed were identified by in situ hybridization and immune-histochemistry, respectively. AtRA is produced from retinol (ROL) in a two step process occurring in nasal epithelium cells. ROL is oxidized into retinaldehyde (RAL), which is then oxidatized into atRA by RALDH3. AtRA diffuses towards the mesenchyme cells, where it activates RARG/RXRA heterodimers anchored on regulatory elements located in *Ahr* gene. In the absence of RALDH3 (*Aldh1a3*$^{-/-}$ mutants) or of RARG (*Rarg*$^{-/-}$ mutants), expression of *Ahr* is reduced, making TCDD unable to induce cleft palate. In addition, atRA may constitute a signal activating heterodimers distinct from RARG/RXRA, and bound to yet unknown atRA-target genes in nasal epithelium cells.

Supplemental Material, References

Abbott BD, Birnbaum LS, and Diliberto JJ. 1996. Rapid distribution of 2,3,7,8-tetrachlorodibenzo-p-dioxin (TCDD) to embryonic tissues in C57BL/6N mice and correlation with palatal uptake in vitro. Toxicol Appl Pharmacol 141:256–263.

Chapellier B, Mark M, Garnier JM, LeMeur M, Chambon P, Ghyselinck NB. 2002. A conditional foxed (loxP-fanked) allele for the retinoic acid receptor alpha (RARalpha) gene. Genesis 32:87–90.

Creech Kraft J, Löfberg B, Chahoud I, Bochert G, Nau H. 1989. Teratogenicity and placental transfer of all-trans-, 13-cis-, 4-oxo-all-trans-, and 4-oxo-13-cis-retinoic acid after administration of a low oral dose during organogenesis in mice. Toxicol Appl Pharmacol 100:162–176.

Dupé V, Matt N, Garnier JM, Chambon P, Mark M, Ghyselinck NB. 2003. A newborn lethal defect due to inactivation of retinaldehyde dehydrogenase type 3 is prevented by maternal retinoic acid treatment. Proc Natl Acad Sci USA 100:14036–14041.

Goelden U, Ukena SN, Pfoertner S, Hofmann R, Buer J, Schrader AJ. (2005). RAR-beta(1) overexpression in chromophobe renal cell carcinoma: a novel target for therapeutic intervention? Exp Oncol 27:220-224.

Hanlon PR, Cimafranca MA, Liu X, Cho YC, Jefcoate CR. 2005. Microarray analysis of early adipogenesis in C3H10T1/2 cells: cooperative inhibitory effects of growth factors and 2,3,7,8-tetrachlorodibenzo-p-dioxin. Toxicol Appl Pharmacol 207:39-58.

Hurst CH, Abbott BD, DeVito MJ, Birnbaum LS. 1998. 2,3,7,8-Tetrachlorodibenzo-p-dioxin in pregnant Long–Evans rats: Disposition to maternal and embryo/fetal tissues. Toxicol Sci 45:129–136.

Hurst CH, DeVito MJ, Setzer W, Birnbaum, LS. 2000. Acute administration of 2,3,7,8-tetrachlorodibenzo-p-dioxin (TCDD) in pregnant Long–Evans rats: Association of measured tissue concentrations with developmental effects. Toxicol Sci 53:411–420.

Lohnes D, Kastner P, Dierich A, Mark M, LeMeur M, Chambon P. 1993. Function of retinoic acid receptor gamma in the mouse. Cell 73:643–658.

Matt N, Schmidt CK, Dupé V, Dennefeld C, Nau H, Chambon P, Mark M, Ghyselinck NB. 2005. Contribution of cellular retinol-binding protein type 1 to retinol metabolism during mouse development Dev Dyn 233:167-176.

Rochette-Egly C, Lutz Y, Saunders M, Scheuer I, Gaub MP, Chambon P. 1991. Retinoic acid receptor gamma : specific immunodetection and phosphorylation. J Cell Biol 115:535–545.

Rochette-Egly C, Plassat JL, Taneja R, Chambon P. 2000. The AF-1 and AF-2 activating domains of retinoic acid receptor-alpha (RARalpha) and their phosphorylation are differentially involved in parietal endodermal differentiation of F9 cells and retinoid-induced expression of target genes. Mol Endocrinol 14:1398-1410.

Roy B, Taneja R, Chambon P. 1995. Synergistic activation of retinoic acid (RA)-responsive genes and induction of embryonal carcinoma cell differentiation by an RA receptor alpha (RAR alpha)-, RAR beta-, or RAR gamma-selective ligand in combination with a retinoid X receptor-specific ligand. Mol Cell Biol 15:6481-6487.

Taneja R, Rochette-Egly C, Plassat JL, Penna L, Gaub MP, Chambon P. 1997. Phosphorylation of activation functions AF-1 and AF-2 of RAR alpha and RAR gamma is indispensable for differentiation of F9 cells upon retinoic acid and cAMP treatment. EMBO J 16:6452-6465.

Vernet N, Dennefeld C, Rochette-Egly C, Oulad-Abdelghani M, Chambon P, Ghyselinck NB, et al. 2006. Retinoic acid metabolism and signaling pathways in the adult and developing mouse testis. Endocrinology 147:96–110.
